# Pancreaticobiliary Malignancies in the Emergency Room: Management of Acute Complications and Oncological Emergencies

**DOI:** 10.1007/s12029-021-00718-7

**Published:** 2021-10-14

**Authors:** Konstantinos Kamposioras, Joe Geraghty, Jordan Appleyard, Mohammed Dawod, Konstantinos Papadimitriou, Angela Lamarca, Alan Anthoney

**Affiliations:** 1 The Christie NHS Foundation Trust, Manchester, Greater Manchester UK; 2 Department of Gastroenterology, Manchester Royal Infirmary, Manchester, UK; 3 St James’ University Hospital, LS9 7TF Leeds, UK; 4 Early Clinical Trials Unit, Antwerp University Hospital, Antwerp, Belgium; 5 Division of Cancer Sciences, School of Medical Sciences, Faculty of Biology, Medicine and Health, University of Manchester, Manchester, UK; 6 Leeds Institute of Medical Research, St James’ Institute of Oncology, St James’ University Hospital, University of Leeds, Leeds, LS9 7TF UK

**Keywords:** Pancreatic cancer, Cholangiocarcinoma, Gall bladder cancer, Emergencies, Complications, Management

## Abstract

**Background:**

Management of pancreaticobiliary (PB) malignancies remains a clinical challenge. In this review, we focus on the management of oncological emergencies in PB malignancies and the potential complication of associated therapeutic interventions.

**Methods:**

Biobliographic review of current evidence on the management of oncological emergencies, their potential complications, as well as synthesis of recommendations was performed. The pathogenesis, frequency, related symptoms as well as appropriate investigations are presented.

**Results:**

The oncologic emergencies in PB patients were summarised in six categories: (1) hematological (including febrile neutropaenia, thrombocytopenia, coagulopathies), (2) gastrointestinal (gastric outlet and biliary obstruction, gastrointestinal bleeding), (3) thromboembolic events, (4) ascites, (5) metabolic disorders and (6) neurologic complications. The pathogenesis, frequency, related symptoms as well as appropriate investigations are also presented.

**Conclusion:**

Patients with PB malignancies are at increased risk of a wide variation of medical emergencies. Clinical knowledge, early recognition and collaboration with the relevant specialties are critical to manage these complications effectively, tailoring overall management around the actual prognosis and individuals’ expectations.

## Introduction 

**Fig. 1:**
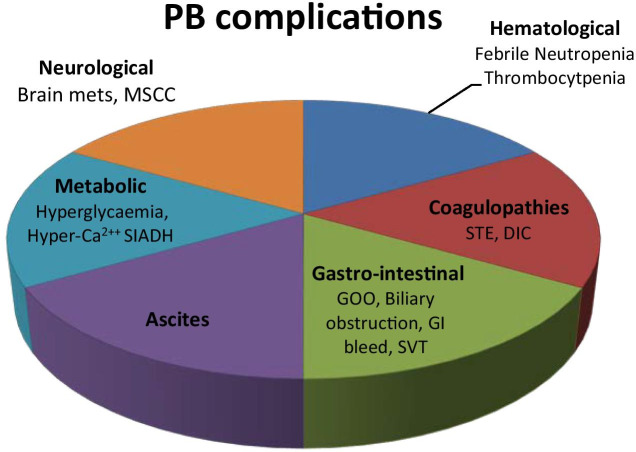
Pancreaticobiliary complications. STE systemic thromboembolic events, DIC disseminated intravascular coagulation, GOO gastric outlet obstruction, SVT splanchnic vein thrombosis, SIADH syndrome of inappropriate antidiuretic hormone secretion, MSCC metastatic spinal cord compression

The management of pancreaticobiliary (PB) malignancies (pancreatic cancer, gallbladder cancer, cholangiocarcinoma and ampullary tumours) remains a significant challenge for clinicians [1]. Late disease presentation, limited response to systemic treatments and patient comorbidities all contribute to the continued dismal survival figures. In addition, patients with PB malignancies may present acutely with specific oncological complications related to features of local disease infiltration, tumour stimulated paraneoplastic conditions or treatment related side effects. Clinicians need to be aware of these to allow provision of appropriate support in a timely manner with optimal utilisation of health resources. This review focuses on key oncological emergencies and acute complications observed in PB malignancies, focusing on their management and the potential complications of associated therapeutic interventions (Fig. 1).

## Hematological

### Febrile Neutropaenia

Febrile neutropaenia (FN), almost always secondary to the effects of chemotherapy, is defined as an oral temperature of > 38.3 °C or two consecutive readings of > 38.0 °C for 2 h and an absolute neutrophil count (ANC) of < 0.5 × 10^9^/L, or expected to fall below 0.5 × 10^9^/L [2]. Although FN can cause significant morbidity and mortality, early diagnosis and aggressive supportive management, particularly commencement of intravenous antibiotics, has significantly improved outcomes with less than 5% of cases in England requiring intensive care management. Use of the internationally recognised Multinational Association of Supportive Care in Cancer (MASCC) prognostic index can help to stratify the clinical management of FN, depending on risk of mortality [3].

#### Frequency

The incidence of chemotherapy-induced FN in PB cancer varies between reported studies but is generally low (Table 1). The use of combination chemotherapy with irinotecan, oxaliplatin and 5FU in metastatic pancreatic ductal cancer produces a high rate of grade 3/4 neutropaenia but sepsis is rare [4]. Although primary G-CSF prophylaxis was not allowed within the trial, 42% of patients on FOLFIRINOX required it as secondary prophylaxis after severe neutropaenia. Modifications of the FOLFIRINOX regimen with, for example, lowering of the irinotecan dose and removal of bolus 5FU, are often made thus reducing the risk of FN.

**Table 1:** Representative phase III studies indicating the frequency of grade 3/4 neutropaenia and FN in PB cancers

	Author (trial)	Line of Tx	Treatment arms	Neutropenia (%) Gr 3/4	FN (%)	Thrombocytopenia (%) Gr 3/4
Pancreatic cancer	Cunningham 2009	1st	Gem/Cap Gem	35 22	4 2	11 6
	Colucci 2010	1st	Gem/Cis Gem	25 14	0 1	16 5
	Conroy 2011	1st	Folfirinox Gem	45.7 21	5.4 1.2	9.1 3.6
	Von Hoff 2013	1st	Gem/nab-Paclitaxel Gem	38 27	3 1	13 9
	Neoptolemos 2017 (ESPAC 4)	Adjuvant	Gem/Cap Gem	38 24	NR	2 2
	Conroy 2018	Adjuvant	Folfirinox Gem	28.4 26.0	3.0 3.7	1.3 4.5
Biliary cancer	Valle 2010 (ABC-02)	1st	Gem/CDDP Gem	25.3 16.6	10 7	8.6 6.5
	Lee 2012	1st	Gem/Ox Gem/Ox/erlotinib	4 2	6 4	0 2
	Sakai 2018	1st	Gem/CDDP Gem/CDDP/S1	48 39	4 5	12 9

Neut: grade 3: < 1000–500/mm^3^; grade 4: < 500/mm3; PLTs: grade 3 < 50,000–25,000/mm^3^; grade 4: < 25,000/mm^3^

*CAP* capecitabine, *CDDP* cisplatin, *FN* febrile neutropaenia, *GEM* gemciatbine, *NR* not reported

Of interest, the incidence of FN observed with gemcitabine (7%) and gemcitabine/cisplatin (10%) regimens used in the ABC-02 study for advanced biliary tract cancers, was higher than observed in pancreatic cancer studies [5]. One explanation for this may be the approximately threefold higher presence of biliary stents, as seen within ABC-02 participants, as opposed to pancreatic studies with the associated increased risk of biliary sepsis.

#### Management

In case of suspected NF, immediate empiric antibiotic treatment should be considered, with a broad spectrum antibiotic (usually beta lactam monotherapy with piperacillin/tazobactam, except when patient-specific or local microbiological contraindications dictate), ideally to commence within one hour of presentation. Thereafter, management can follow risk stratification. High-risk patients (MASCC < 21) should be admitted to continue on intravenous antibiotics and other supportive measures. Low-risk group (MASCC ≥ 21, haemodynamically stable, without evidence of organ failure, pneumonia, an indwelling venous catheter or severe soft tissue infection) can be treated with antibiotics such as combination quinolone with amoxicillin plus clavulanic acid [6] or oral moxifloxacin, as an outpatient [7].

The use of granulocyte colony-stimulating factors (G-CSFs) in treatment of chemotherapy-induced FN has not been shown to improve overall survival but can induce a faster recovery from fever and shorter hospital stay [8]. Use of G-CSF to prevent recurrence of FN (secondary prophylaxis) is common with FOLFIRINOX with occasional use as primary prophylaxis.

### Thrombocytopenia

Thrombocytopenia is, in itself, not recognised as an oncologic emergency but chemotherapy regimens associated with treatment of PB malignancies do have a high incidence of grade 3 (< 50.0–25.0 × 10^9^/L) and 4 (< 25.0 × 10^9^ /L) toxicity. This can be up to 40% (12% grade 4) with certain combinations of gemcitabine and cisplatin [9] but in the registration studies of the most commonly used regimens was around 10%, with rates of the more clinically concerning grade 4 toxicity not individually reported [4, 5, 10]. The management of thrombocytopenia is driven by any bleeding symptoms aiming to maintain the platelet level above 10.0 × 10^9^/L unless there are additional risks for bleeding [11].

### Coagulopathies

#### Systemic Thromboembolic Events

##### Frequency

PB cancers are known to be associated with an increased risk of thrombotic events. A 6-year study of pulmonary embolism (PE) incidence in cancer outpatients showed PB cancers accounting for almost 13% of all detected PE with a relative risk of 2–2.5 × that of the average cancer patient [12]. Thrombosis can be the first manifestation of an otherwise undiagnosed cancer and PB cancers may account for 15% of cancer associated PE/DVT presenting in this manner [13].

Up to 60% of venous thromboembolic events (VTEs) in PB cancer patients may present asymptomatically [14]. Alternatively, common presenting manifestations of acute peripheral deep vein thrombosis (DVT); limb oedema, pain, erythema, or of PE; breathlessness, chest pain and tachypnoea may be masked in cancer patients by other features of their disease.

Arterial thrombosis may also be increased in PB cancer. An analysis from 4 stroke units in France over a 7-year period recorded 17 cases of ischaemic stroke in patients with pancreatic cancer [15]. In 7 of these patients, the onset of the stroke led to the cancer diagnosis. Non-bacterial thrombotic endocarditis was detected in about a third of the patients by cardiac ultrasound. Outcome was very poor for all patients irrespective of anticoagulation.

##### Investigations

Predictive clinical models, such as the Wells or revised Geneva score, may help in assessing the risk of VTE in cancer patients [16]. D-dimer levels in PB cancer patients may be elevated as part of the hypercoagulable state, even in the absence of VTE, resulting in a threefold higher rate of false positives. However, if not elevated they can help in exclusion of VTE in cancer patients and save further investigations.

Duplex ultrasonography of limb veins remains the principle diagnostic test for DVT. CT or MRI can be used for looking at deep pelvic veins. CT pulmonary angiography is routinely used to detect PE with a high sensitivity, and negative predictive value > 94% [17].

##### Aetiology

Notable advances have been made over recent years in our understanding of the molecular mechanisms underlying cancer related thrombosis. Tissue Factor, a transmembrane protein found normally in sub-endothelial tissue [18], cancer associated mucins and the protein podoplanin expressed in PB cells [19] can stimulate the extrinsic coagulation pathway and activate platelets to stick to endothelial cells and leukocytes resulting in formation of micro-thrombi. Tumour-associated activated neutrophils release histone-bound DNA fragments with associated granular proteins. Although these neutrophil extracellular traps (NET) have ostensibly an immune protective function they contribute to the pro-coagulant environment by capturing activated platelets and micro-vesicles [20].

Patient-related factors also contribute to the risk of thrombosis development. The administration of certain cytotoxic drugs, e.g., platinums, is known to have a pro-coagulant effect, as seen by the increase in VTE events in patients on chemotherapy compared to those not on treatment [21]. However, exact mechanisms underlying this remain unclear. Similarly, indwelling venous catheters to deliver chemotherapy are associated with an increased risk of venous thrombosis [22].

##### Management

Low molecular weight heparins are a more effective first-line treatment compared to vitamin K antagonists such as warfarin [23]. The optimal duration of anticoagulation remains unproven; however, recent evidence suggests that continuing anticoagulation, of whatever form, for over 6 months had a significant benefit in terms of reduced incidence of further VTE than only being on treatment for 0–3 months [24]. Many patients find long-term self-administration of LMWH problematic, and the direct oral anti-coagulants (DOACs) have become an alternative first-line treatment for cancer-related VTE. Large, randomised, non-inferiority trials of edoxaban, rivaroxaban and apixaban compared to LMWH in cancer-induced VTE have shown equivalence in terms of reducing VTE recurrence [25–27] (Table 2), with small but significant increases in the risk of clinically relevant bleeding and more frequent drug-drug interactions needing to be taken into consideration. As experience becomes greater with the new agents, it may be that the type of anticoagulant used during hospitalisation and out-of-hospital periods for cancer patients with VTE will vary.

**Table 2:** Treatment of cancer-related venous thromboembolic event (VTE)

Trial	Direct oral anticoagulant	Patient number	Recurrent VTE	Major bleed	CRNMB	Fatal bleed
Carravagio [27]	Apixaban 10 mg bd for 7 days, then 5 mg bd	576	5.60%	3.8% (1.9% GI)	9.00%	0
	Dalteparin 200 IU sc. daily for 1 month then 150 IU sc. daily	579	7.90%	4.0% (1.7% GI)	6.00%	2
	Treatment for 6 months					
Hokusai VTE [25]	Edoxaban 60 mg/day	522	7.90%	6.90%	14.60%	
	Dalteparin 200 IU sc. daily for 1 month then 150 IU sc. daily	524	11.30%	4.00%	11.10%	
	Treatment for at least 6 months–extension to 12 months at medical discretion					
SELECT-D [26]	Rivaroxaban	203	4%	5.50%	12%	
	Dalteparin 200 IU sc. daily for 1 month then 150 IU sc. daily	203	9%	3%	3.50%	

*CRNMB* clinically relevant non-major bleeding

A number of trials looking at the role of prophylactic LMWH and DOACs in pancreatic cancer, or where pancreatic cancer formed a large proportion of the trial cohort, have been published [28, 29] (Table 3). They show significant reductions in the risk of VTE, both incidental and symptomatic, but as yet it is unclear as to whether they impact on overall survival. Our greater understanding of the molecular basis underlying PB cancer hypercoagulability may ultimately allow patient tailored prophylactic anticoagulation therapy in future.

**Table 3:** Prophylaxis for cancer-associated venous thromboembolic event

	Direct oral anticoagulant	Patient number	Khorana score 2/ > 2	VTE (DVT/PE)	Major bleed	CRNMB
Cassini [29]	Rivaroxaban 10 mg daily for 180 days	420	286	24 (15/5)	8	11
	Placebo	421	298	37 (19/5)	4	8
Avert [28]	Apixaban 2.5 mg bd for 180 days	291	63.9/26.1	12 (7/5)	10	21
	Placebo	283	67.1/23.9	28 (12/16)	5	15

*CRNMB* clinically relevant non-major bleeding

### Disseminated intravascular coagulation (DIC)

The most extreme manifestation of activation of the coagulation system is disseminated intravascular coagulation (DIC). Overt DIC is uncommon in PB malignancy but has a very poor prognosis.

#### Symptoms

The commonest form of DIC in PB malignancy is ‘procoagulant’, where excess thrombin generation causes both microvascular and macrovascular thrombosis. However, the hyperfibrinolytic form, where an increased risk of bleeding due to activation of the fibrinolytic system is dominant, has also been reported in pancreatic cancer [30]. At its most extreme patients can present with multiple thromboses in peripheral or visceral vessels, bleeding with associated thrombocytopenia or a combination of both.

#### Investigations

Interpretation of coagulation blood tests can be challenging in cancer-associated DIC. The prothrombin time (PT) and partial thromboplastin time (PTT) may not be prolonged in subclinical DIC, and paradoxically the PTT can even be decreased initially [30, 31]. Fibrinogen levels are not usually altered in pro-coagulant DIC but may be significantly reduced in hyperfibrinolytic disease.

#### Management

There is no clear consensus regarding the optimal management strategy; however, patients should be assessed to identify those with high risk of bleeding and those with high risk of thrombosis [30, 31]. Precipitating factors, such as sepsis, should be considered and treated if present. Appropriate treatment of the cancer is considered to be the first-line strategy for cancer-related DIC. Anticoagulation with therapeutic-dose of low molecular weight heparin (LMWH) for 6 months, especially when thrombosis is clinically overt, in the presence of satisfactory platelets counts, is safe and superior to warfarin in preventing recurrence of thromboembolic events [31, 32]. Platelets and plasma support showed proven efficacy in patients with low platelets and bleeding risk. Other measures include concentrate of anti-thrombin III inhibitor and supplementation of activated protein C.

#### Complications of Management

Bleeding is a known complication of DIC treatment especially if anticoagulation monitoring is suboptimal. Increased risk of clotting is also noted in patient receiving clotting factors to correct coagulopathy. Other less common complication associated with transfusion reactions.

## Gastrointestinal

### Gastric Outlet Obstruction and Functional Gastroparesis

#### Frequency

In pancreaticobiliary cancers, gastric outlet obstruction (GOO) occurs in around 5% of patients at presentation with a further 10–20% of patients developing GOO during the course of the disease [33–36]. Gastroparesis, a disorder characterised by delayed emptying of gastric contents with resulting nausea, vomiting and upper abdominal fullness, is potentially even more common in PB malignancy. Reported as occurring in 60% of patients with pancreatic cancer [37], half of whom showed symptoms, causes may relate to tumour infiltration into autonomic neural plexuses, medication or, rarely, paraneoplastic phenomena.

#### Symptoms

GOO may present acutely with nausea and vomiting which may be projectile and large volume with undigested food residue. Patients often have a feeling of epigastric fullness or discomfort which is relieved by vomiting. If left untreated, it may lead to dehydration and cachexia with repeated episodes of vomiting risking aspiration pneumonia. However, GOO-like gastroparesis can also develop insidiously with early satiety, decreased appetite and weight loss, worsening reflux symptoms and severely reduces the patients’ quality of life (QoL) [36].

#### Investigations

Unlike small intestinal obstruction, features of GOO may not be obvious on abdominal X-ray despite similarities in acute symptoms. CT imaging is the mainstay of diagnosis allowing identification of tumour location and its relationship to adjacent structures. It is important to look for secondary sites of obstruction beside the primary PB tumour itself. This can include locoregional lymph nodes and peritoneal disease involving distal segments of bowel. If peritoneal disease is present, good palliation is much harder to achieve. For more accurate information on the extent of obstruction and to plan effective treatment, a barium meal or gastroscopy may be helpful.

If no obvious site of mechanical obstruction is determined despite symptoms, a gastric emptying study may be required to identify gastroparesis. It is an important diagnosis to make in view of its prevalence, up to 60% of PB malignancies, and being misattributed as cancer cachexia or chemotherapy-induced nausea and vomiting.

#### Management

The treatment goal is to reestablish oral feeding and resolve symptoms by restoring gastrointestinal continuity. Operative resection is the best treatment if the cancer is potentially resectable. If at the time of laparotomy, the tumour is found to be unresectable, then operative bypass should be performed. This is because there is a 28% risk of developing GOO during follow-up [38].

For patients with upfront unresectable disease, there is no consensus for treatment of malignant GOO. Since the 1990s, the traditional, open gastrojejunostomy (OGJ) has been replaced by either endoscopic duodenal stenting (DS) or laparoscopic gastrojejunostomy (LGJ) [36]. DS is associated not only with a shorter length of stay (LOS) and faster symptom relief but also with an increased risk of symptom recurrence and need for reintervention [35, 39, 40]. Most DS procedures can be performed as an outpatient procedure, and patients can introduce a soft diet from the following day. For patients undergoing LGJ, oral diet can be reintroduced between 4 and 10 days post-op [41]. A systematic review showed that the clinical success was 89% for DS and 72% for LGY [42].

The decision between DS and LGJ should be based on performance score, comorbidities and nutritional status (all of which if impaired should favour DS), while longer predicted survival favours LGJ. It is important to remember that the mean survival times after a diagnosis of GOO is only 7 to 20 weeks [35, 43]. Many commentators now believe that DS achieves a better palliative result for most patients. This is because it minimises pain, time in hospital and physiological stress, while not differing from LGJ in terms of survival or overall complications [39]. The exception to this would be patients with a high performance score undergoing combination chemotherapy such as FOLFIRINOX with a longer predicted survival time.

**Fig. 2:**
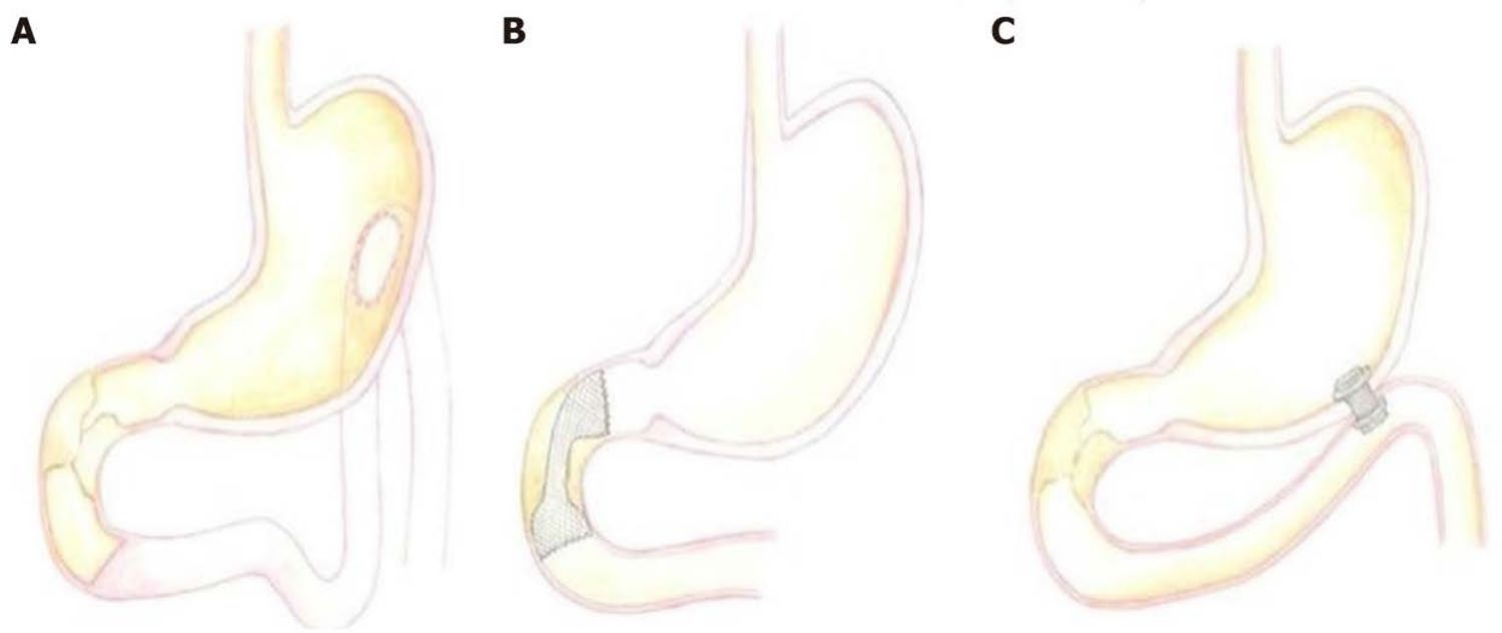
The three current approaches for GOO. **A** Laparoscopic gastrojejunostomy (LGJ). **B** Endoscopic duodenal stenting (DS). **C** Endoscopic gastrojejunostomy ( adopted from Troncone E, Fugazza A, Cappello A, Blanco GDV, Monteleone G, Repici A, Teoh AYB, Anderloni A. Malignant gastric outlet obstruction: which is the best therapeutic option? *World J Gastroenterol* 2020; 26(16): 1847–1860)

A third alternative option for palliation is rapidly emerging. The use of endoscopic ultrasound (EUS) and lumen apposing metal stents to create an endoscopic gastrojejunostomy. Early data, which needs replicating, suggests the technique may provide benefits over both alternatives and reduce complications [44] (Fig. 2). It may also be of benefit when altered anatomy or severe proximal duodenal obstruction precludes standard stenting options.

If nausea and vomiting occur in the absence of mechanical obstruction, then malignant gastroparesis should be considered. Initial treatment should include review of potentially contributing medications such as calcium channel blockers, opioid analgesics and tricyclic antidepressants, and any coexisting diabetes should be optimised. The next step is usually to offer dietary and behavioural modifications including having small, frequent, low-fat, low-residue meals. Prokinetics and antiemetics form the mainstay of pharmacological therapy and the treatments are the same as for non-cancer gastroparesis. Erythomycin appears the most potent agent for stimulating gastric emptying when compared with domperidone, metoclopramide or cisapride [45].

#### Complications from Management (Procedures)

The significant early complications from DS include bleeding and perforation, occurring in around 1% of cases. Longer term, tumour ingrowth or stent migration has been reported in up to 23% of cases [46]. Late complications occurred in 17% of LGJ including anastomotic leakage and dysfunction of the LGJ [42].

### Biliary Obstruction

#### Frequency

Approximately 70% of patients with pancreatic cancer present with Malignant Biliary Obstruction (MBO) [47]. Cholangiocarcinoma and gallbladder cancer also commonly cause obstruction through direct compression, hilar node spread or metastatic disease. Ampullary cancer while rare almost always causes MBO.

#### Symptoms

MBO leads to painless jaundice, pruritus, pale stools, dark urine, lethargy, anorexia, nausea and vomiting.

#### Investigations

Blood tests will confirm raised (conjugated) bilirubin along with other cholestatic liver enzymes. Tumour markers such as carbohydrate antigen 19–9 (CA 19–9) will also be raised, but this can occur in benign causes of biliary obstruction and is seldom clinically useful [47]. Liver ultrasound can help identify biliary obstruction and whether intra-hepatic or extra-hepatic. However, it is often not sufficient by itself to identify the cause and guide subsequent management and, in most cases, a CT is required to provide cross-sectional imaging of the obstruction.

When MBO occurs de novo radiological investigations need to accurately stage the causative disease. Appropriately protocolled CT can help define resectability by describing local and distant disease, with CT angiography evaluating invasion or involvement of regional arteries and veins. In MBO arising at the hilar and intrahepatic regions, further anatomical assessment is often required. The most accurate modality in this case is magnetic resonance cholangiopancreatography (MRCP), with a sensitivity exceeding 96% and a specificity of 85% for distinguishing benign from malignant biliary obstruction. It also allows procedural planning for resectability and in palliative cases provides a road map for biliary drainage, for example by helping determine whether endoscopic or percutaneous approaches are feasible. [47].

#### Management

If the lesion is resectable and the patient fit enough, then the optimal biliary drainage approach is surgical resection. The issue of preoperative biliary drainage (PBD) has been controversial for many years been. A large recent meta-analysis suggested that avoiding PBD as a default position is associated with better outcomes. Notable exceptions include presence of cholangitis or intractable pruritus [47]. Decisions based on a bilirubin level remain contentious and while a level of 250 μmol/l has been advocated as requiring PBD a better more practical approach is to offer drainage if surgery is likely to be delayed (> 2 weeks), and in patients undergoing neoadjuvant chemotherapy. This later group is becoming increasingly common with the promotion of novel regimes, such as FOLFIRINOX.

In non-resectable disease, ERCP is the first-line approach to biliary drainage for all but a subsection of perihilar lesions. It is preferred to surgery because operative morbidity and mortality is high (up to 25% in some series) [48]. Compared to PTC, it is associated with lower rate of adverse events (8.6 vs 12.3%), as well as fewer repeat procedures, shorter hospitalisation, lower costs and the avoidance of external drainage catheters [48]. When obtaining biliary drainage the best approach is to use self-expanding metal stents (SEMS), instead of plastic stents, as these are proven to be associated with longer patency, lower complication rates, fewer reinterventions and longer patient survival [49, 50].

In approximately 10% of cases, ERCP may not be possible, for example due to proximal duodenal obstruction or altered anatomy post-surgery. In this situation, the emerging role of EUS-guided extra-luminal stenting has emerged as a useful tool to drain the biliary tree via the stomach (hepaticogastrostomy (HG)) or duodenum (choledochoduodenostomy (CD)) [51].

**Fig. 3:**
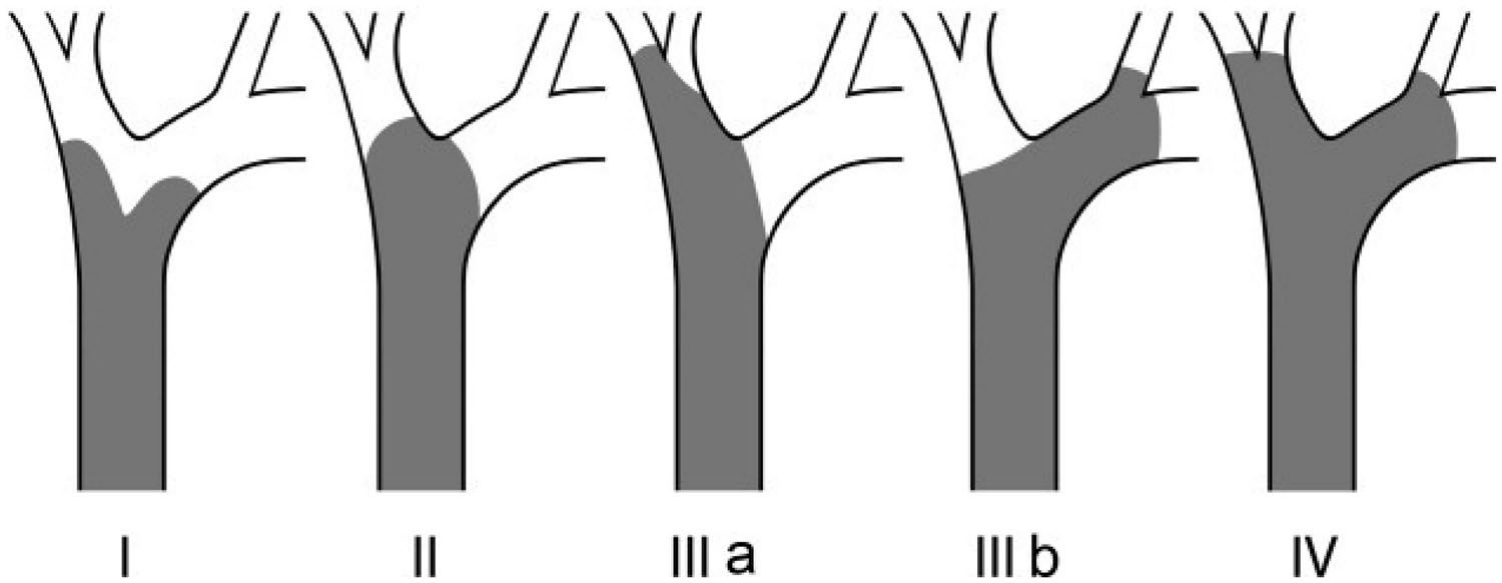
The Bismuth classification for perihilar malignant obstruction (courtesy of Professor Henri Bismuth, Paris, France)

In cases of perihilar obstruction, typically related to cholangiocarcinoma, gallbladder malignancy or nodal disease, management decisions are a little more complex. For patients with Bismuth I or II tumours, ERCP is still preferred to PTC. However, patients with more advanced hilar obstruction (Bismuth III or IV) are more difficult to palliate with ERCP (Fig. 3). A retrospective review demonstrated higher success rates with PTC (93% vs 77%), though median survival was similar. Thus, PTC is generally favoured over ERCP in these cases [52].

#### Complications from Management/Procedures

Following ERCP, the most common and potentially serious complication is pancreatitis, occurring in approximately 5% of procedures. Less common complications include perforation, biliary sepsis and bleeding complications from sphincterotomy [53]. Major complications from PTC occur in around 2 to 10% of cases, and include sepsis, liver abscess, bile leak, haemorrhage (subcapsular hematoma and pseudoaneurysm) and pneumothorax [54]. The presence of a biliary stent increases the chance of cholangitis, either biochemical or infective, with associated fever, pain and transient elevation of bilirubin and liver enzymes on blood tests.

### Gastrointestinal Bleeding

#### Frequency

Around 3% of pancreatobiliary cancer patients experience gastrointestinal bleeding (GIB) [55]. This usually arises from tumour infiltration into the highly vascularised duodenum or, more rarely, the bile duct, stomach, jejunum or colon. The rare occurrence of tumour haemorrhage via the pancreatic duct is termed Haemosuccus Pancreaticus (HP). Bleeding can also occur from gastric varices secondary to splenic vein occlusion [56]. GIB may also occur from benign causes, e.g. peptic ulcer disease and oesophagitis.

#### Symptoms

Symptoms of a GIB include melaena, haematemesis, abdominal distention and pain. Similarly, a fall in haemoglobin accompanied by a rise in urea (out of proportion to creatinine) is suggestive [55]. The extent of bleeding may range from the occult to rapid exsanguination, and this will dictate the speed and scope of investigations.

#### Investigations

Investigations are as for any patient with a GIB, with the possible exception of earlier use of cross-sectional angiography, including clinical examination with digital rectal exam to identify melaena, blood tests (including clotting and platelet count) and once the patient has been appropriately resuscitated, early gastroscopy. In one study, 65% of patients with a GIB and pancreatic cancer actually had peptic ulcer disease (not directly linked to the tumour) [55]. One important caveat is that in cases of HP, bleeding will be through the ampulla of Vater, and this is not always appreciated with standard gastroscopy. For this reason, full assessment requires the use of a side viewing gastroscope (as used in ERCP procedures) to definitely visualise the ampulla [57]. In cases of negative endoscopy and ongoing GIB then triple phase CT often leading to angiography is the gold standard for diagnosis and therapy.

#### Management

Management is determined by the underlying cause. In the case of duodenal infiltration, transcatheter arterial embolisation (TAE) and endoscopic or surgical endoscopic hemostasis are potential treatment options. TAE is more appropriate for arterial embolisation, while surgical hemostasis may increase complications and does not improve the prognosis [58]. Radiotherapy in case of endoscopic haemostasis failure can be an effective option [59, 60]. The evidence for the use of tranexamic acid in the control of GI bleeding is limited [61], while its role in PB cancers is unclear.

When the cause is HP, then the mainstay of treatment is coil embolisation of the accompanying pseudoaneurysm (usually the splenic artery). In cases of gastric variceal bleeding; initial control can be attempted with endoscopic glue injection but if ongoing then splenectomy, splenic artery embolisation and stenting of the splenic vein are the current treatment strategies [62].

#### Complications from Management/Procedures

Endoscopic management of peptic ulcer-associated bleeding is generally safe however recurrent bleeding may occur in up to 10% of cases [63]. Percutaneous arterial embolisation may be complicated by postembolisation hepatic abscesses or failure and rebleeding can occur in up to 25% of cases [64].

## Thrombosis

### SVT and Malignancy-Related Portal Hypertension

#### Aetiology

The anatomical relationship of the splanchnic venous drainage (portal, splenic and mesenteric) to the pancreas, liver and bile duct make them particularly prone to tumour related obstruction or occlusion (splanchnic vein thrombosis (SVT)). This can arise through extrinsic compression of vessels directly by tumour or lymph node metastases, by tumour induced thrombosis or by tumour invasion into the vessel. The resultant local blood flow stasis and endothelial injury, along with the hypercoagulable state observed in PB cancers, comprises the classical Virchow’s triad of cancer-related thrombosis.

#### Epidemiology

Although there are non-malignant causes of SVT, it is strongly associated with malignancy. This association is sufficiently clear for some to suggest that development of SVT in patients without liver cirrhosis is a marker for occult malignancy with an 8% absolute cancer risk at 3 months [65]. In pancreatic cancer and cholangiocarcinoma, the increased risks of development of SVT are significant at between 30 to 75 times the general population risk, respectively [66].

#### Presentation

Portal vein thrombosis (PVT) can be classified depending on position (main trunk, branch or both), being acute or chronic and whether symptomatic or asymptomatic [67].

In PVT, it is known that venous collaterals develop within 3–5 weeks of the acute event. Therefore, the definition of acute and chronic PVT to some extent depends on symptoms and the radiological presence of collaterals. Many cases of malignancy associated PVT arise without symptoms and are picked up as incidental features on imaging. In addition, the features of acute PVT are similar to symptoms commonly seen in PB cancer, such as abdominal pain, fever, nausea, anorexia and early satiety. Chronic PVT is characterised by the development of local venous collaterals, the ‘portal cavernoma’, and portal hypertension with associated risks of gastrointestinal (GI) haemorrhage and hypersplenism. In a study of PVT occurring in 118 patients with predominantly pancreatico-biliary cancers, 38% had radiological features of portal hypertension and 18% had a GI bleed during the follow up period (median 10 months) [68]. Oesophageal varices were the most common source of bleeding; however, less common sites of varices, e.g. duodenum, gall bladder, are seen particularly when thrombus involves more distal branches of splanchnic venous system.

Hypersplenism develops in acute and chronic PVT, as well as in splenic vein thrombosis. Splenomegaly is usually detectable and reduced circulating leukocytes and platelets, secondary to sequestration, is observed.

Mesenteric vein thrombosis (MVT) can present variably, e.g. acutely, with onset of severe abdominal pain out of proportion to physical findings, or more chronically with abdominal pain, diarrhoea and weight loss. MVT is associated with features of mesenteric ischaemia in up to 15% of cases.

Thrombosis or occlusion of the splanchnic venous drainage can be associated with development of ascites. In this situation, the ascitic fluid is a transudate (low serum/ascitic fluid protein ratio) with few or no malignant cells. In some cases, it can be large volume, rapidly reaccumulating and causes significant morbidity.

#### Diagnosis

Many instances of PVT are picked up incidentally on CT in patients with no specific symptoms. In patients with concerning symptoms Doppler ultrasound is a highly sensitive (~ 90%) and specific (~ 95%) initial test [66]. Differentiating between tumour and bland PVT relies upon specific features on contrast enhanced CT or MRI or use of ^18^F-FDG PET. CT and MRI are also required for diagnosis of MVT.

#### Treatment

There is no specific prospective clinical trial data relating to the use of anticoagulation in cancer related SVT. International guidelines on management for portal vein thrombosis, of any aetiology, generally recommend long-term anticoagulation in the presence of a persisting predisposing stimulus, such as cancer [69]. In general, the potential benefits of anti-coagulation in terms of vessel recanalisation (in acute SVT), thrombus progression and development or deterioration of symptoms have to be balanced with risks of toxicity. This is particularly with patients with portal hypertension and varices or other bleeding risks. Although DOACs can be used in cancer-related thrombosis, careful consideration has to be made on a patient by patient basis with regard to interactions with other medications and reversibility of anticoagulation in event of a major GI bleed [70].

Percutaneously inserted self-expanding metal mesh (SEMM) endoluminal vascular stents can be used to open occluded portal and mesenteric veins to reduce symptoms arising from portal hypertension. In a study of 22 patients with pancreaticobiliary cancers and GI bleeding or ascites, placement of stents resolved symptoms in all but 2 patients [71]. Procedural complications were rare, but reocclusion occurred in 25% of patients. Patient selection may be important as a significant difference was observed in post-stent overall survival depending on the palliative prognostic index score in another study [72].

## Ascites

### Frequency

Malignant ascite (MA) is a common manifestation of advanced pancreatic, and less frequently biliary, cancer. In an analysis of 180 patients with pancreatic cancer who developed ascites, the median time to onset of ascites from diagnosis was 11 months, with median survival following this 1.8 months [73].

### Pathophysiology

The pathophysiology of ascites in PB cancers is multifactorial. Firstly, splanchnic vessel occlusion can result in extra-hepatic portal hypertension and activation of the renin–angiotensin–aldosterone system, resulting in water and sodium retention [74]. Secondly, animal studies suggest lymphatic obstruction as a possible underlying mechanism, although lymphatic blockage alone cannot explain how some patients, with small tumour burdens, experience large fluid accumulation. More recently, vascular permeability factors, immunomodulators and metalloproteinases have gained increasing interest for their role in the formation of MA [75]. Vascular endothelial growth factor (VEGF) levels are significantly increased in patients with MA compared to patients with non-malignant cirrhotic ascites used as controls, thus increasing the permeability of endothelial cells [76].

### Management

#### Diuretic Therapy

Use of diuretics is of symptomatic benefit in less than 50% of patients with PB cancer associated ascites [77] although the underlying aetiology can help guide which patients might benefit more. Patients whose ascites is the result of portal hypertension, secondary to liver metastases or portocaval adenopathy, have been shown to lose up to twice as much fluid per day than those where peritoneal carcinomatosis is the underlying cause, when treated with spironolactone or furosemide [78]. They also had fewer problems with third space effects such as hypotension or renal dysfunction.

#### Ascitic Drainage and Cell-Free and Concentrated Ascites Reinfusion Therapy

Paracentesis is often considered to be the mainstay treatment for MA. However, its effects can be short-lived with patients requiring repeat paracentesis, sometimes every few days [79]. Concerns around infection, particularly bacterial peritonitis, restrict such acute parcentesis to being done in hospital. In patients requiring frequent ascitic drainage, semi-permanent tunneled catheter drainage systems, e.g. PleurX®, may be of benefit. The catheter is inserted into the peritoneal cavity via a subcutaneous tunnel, to help protect against infection, and then connected to a vacuum bottle via a drainage line. Patients are able to self-perform fluid drainage at home without the need to visit hospital, which consequently leads to better management of symptoms, such as shortness of breath and abdominal pain [80]. As ascitic fluid is rich in proteins such as albumin and globulin repeat paracentesis leads to a loss of body protein which can result in symptoms such as fatigue. Cell-free and concentrated ascites reinfusion therapy (CART) takes the fluid from paracentesis, filters out any cellular debris and concentrates the fluid protein before reinfusing it back into the patient [81]. Hanada et al. analysed CART on 51 patients with various malignancies (16% diagnosed with pancreatic cancer) and found that the median time to repeat paracentesis was 27 days [82]. There was also a significant improvement in the intensities of all seven symptom modalities reported: fatigue, abdominal pain, abdominal distention, lack of appetite, nausea and vomiting, shortness of breath and insomnia.

#### Monoclonal Antibody Treatments

VEGF binding monoclonal antibody therapies such as bevacizumab and aflibercept have been shown to reduce the formation of malignant ascites in ovarian and colorectal cancer studies. In a phase II study, Colombo et al. investigated the use of aflibercept on MA in 16 patients with epithelial ovarian cancer [83]. They showed that the repeat paracentesis response rate was 62.5% and that the median time to repeat paracentesis was 4.5-fold greater with aflibercept at 76.0 days than baseline at 16.8 days. As VEGF is a cause of ascitic fluid production in PB cancer, such approaches may also have relevance.

Catumaxomab is a monoclonal antibody which targets epithelial tumour cells expressing epithelial cell-adhesion molecule (EpCAM), as well as T cells via CD3. It also activates the Fcγ-receptor by its Fc domain [84]. In a phase II/III trial, patients with ovarian and non-ovarian cancer (9% pancreatic cancer) were randomised to catumaxomab with paracentesis or paracentesis alone. Both median puncture-free survival and median time to repeat paracentesis were significantly longer in the catumaxomab group at 46 days and 77 days, versus 11 days and 13 days in the control, respectively [84].

## Metabolic Disorders

### Hyperglycaemia and Diabetic Ketoacidosis

#### Aetiology

Pancreatic adenocarcinoma has a strong association with hyperglycaemia with up to 80% of patients with pancreatic cancer recognised as having impaired glucose tolerance or clinical diabetes mellitus (DM) [85]. Although it is possible that DM may act as a risk factor for development of pancreatic cancer, the exact relationship at a pathophysiological level is unclear. Pancreatic atrophy and disrupted blood flow due to tumour bulk may reduce islet cell number and function with reduced insulin secretion.

#### Presentation and Management

In most patients with pancreatic cancer, the manifestation of DM will be poor glycaemic control. However, occasionally patients will present with diabetic emergencies—particularly diabetic ketoacidosis (DKA). In reports of pancreatic adenocarcinoma presenting as DKA, patients have had a prior history of type 2 DM, which normally rarely causes DKA, and some associated precipitating factor such as pancreatitis or infection that may have been stimulated by the occult pancreatic cancer [86]. Further investigation of the patient revealed the pancreatic malignancy. Diabetic ketoacidosis is also associated with pancreatic glucagonoma and somatostatinoma. Although these tumours are very rare, they should be considered in the differential diagnosis. The slow growing nature of these tumours may result in repeat presentations with DKA without other particular symptoms [87].

The acute management of DKA across the world is standard. Reference to national or local guidelines is recommended.

### Hypercalcaemia

#### Frequency

Hypercalcaemia, defined as a serum adjusted Ca^2+^above 2.6 mmol/L, is a well-recognised cancer-associated problem, and although rarely observed in PB cancer, it may be more common in pancreatic neuroendocrine tumours [88].

#### Aetiology

Tumour production of parathyroid hormone-related polypeptide (PTH-rp) increases renal tubular calcium reabsorption and phosphate excretion, as well as acting on osteoblasts [89]. It can arise with extensive bone metastases, where release of tumour cytokines results in increased osteoclast activity and enhanced bone resorption.

#### Symptoms

Symptoms of hypercalcaemia depend on the plasma Ca^2+^ level and include fatigue, muscle pain and weakness, anorexia and constipation. Severe hypercalcaemia can result in delirium, stupor, coma, cardiac arrhythmia and renal failure [89].

#### Investigations

Serum biochemical assessment to confirm raised serum adjusted Ca^2+^ (normal range 2.2–2.6 mmol/L), as well as renal function in case of dehydration and to guide bisphosphonate dosage. Mid-morning parathyroid hormone (PTH) level may be required to exclude endocrine related hypercalcaemia (usually low in malignancy-related hypercalcaemia). PTH-rp can also be measured and is highly specific for malignancy-related hypercalcaemia [90].

#### Management

This is guided by the level of serum calcium and symptoms. Eliminate iatrogenic causes of hypercalcaemia (Ca ^2+^ and Vit D3 supplements, thiazide diuretics). If evidence of dehydration and renal impairment administration of IV fluids is advised. Bisphosphonates, e.g. zoledronic acid, should be administered concurrently with IV fluid as the hypocalcaemic effect starts within 2–4 days.

Calcitonin can be used given its prompt effect and favourable side effect profile; however, the response is short lived. Patients with refractory hypercalcaemia should be considered for denosumab, an inhibitor of the receptor activator of nuclear factor κB ligand (RANKL). Cinacalcet (a calcimimetic agent which directly lowers PTH levels) should be considered in patients with resistant hypercalcemia as a result of PTH over production [91].

#### Complications from Management

During the rehydration, it is important to monitor fluid balance and ensure that patient is not developing overload. Osteonecrosis of the jaw is a known side effect from bisphosphonates and RANK treatment. Dental history and examination are important before proceeding with treatment.

### Syndrome of inappropriate antidiuretic hormone secretio (SIADH)

#### Frequency

The syndrome of inappropriate ADH (SIADH) production is the most common cause of euvolemic hyponatraemia and in cancer patients can be caused by ectopic ADH secretion. SIADH in pancreaticobiliary malignancies is very uncommon with, to date, a total of 13 case reports of documented ectopic ADH production in pancreatic and pancreatic neuroendocrine tumours.

#### Clinical Presentation

SIADH presents with hyponatraemia and symptoms are related to severity and acuteness of onset. The concern is regarding fluid shifts resulting in cerebral oedema. This may be suggested in patients with new onset moderate, or worse, hyponatraemia (Na^+^  < 130 mmol/L) presenting with symptoms ranging from headache, nausea and vomiting to bradycardia, hypertension, convulsions and coma. Conversely, patients with chronic hyponatraemia are often asymptomatic.

#### Investigations

Checking renal function, serum Na ^+^ (S.Na ^+^), serum osmolality and urinary Na ^+^ and osmolality will provide adequate information to diagnose SIADH. Cortisol and thyroid function test can help to exclude other possible causes of hyponatremia.

#### Management

Mainstay of treatment is to control the underlying malignancy. Fluid restriction to 500 ml/day is helpful in mild cases, and effect may be transient in nature. For severe and symptomatic hyponatremia, hypertonic saline is recommended, aiming to increase S.Na^ +^ by 0.5–1 meq/l/h; symptoms improve with 4–5% correction of the S. Na ^+^ . Appropriate endocrinology advice is crucial in treating cancer-related hyponatremia and discuss about starting tolvaptan, an oral elective vasopressin V2-receptor antagonist that is approved for euvolemic and hypervolemic hyponatremia [92].

#### Complications from Management

Central pontine myelinolysis is a known side effect of fast correction of hyponatremia, manifested by sleepiness, hallucination, confusion tremors, slurred speech and weakness.

## Neurological Emergencies

### Metastatic Spinal Cord Compression (MSCC)

#### Frequency

Metastatic spinal cord compression (MSCC) is an oncological emergency that needs urgent management. More than 50% of MSCC diagnosis is associated with prostate, lung or breast cancer. In patients with GI cancer including hepatobiliary MSCC at diagnosis or during the course of disease is rare (2%).

#### Symptoms

Pain is the most common presentation in > 90% of cases. This can be local or radicular in nature, often described as a new pain or change in character of previous pain. The motor, sensory and autonomic signs occur late in the evolution of MSCC and will be distributed below the level of the cord compression and associated with poor outcome.

#### Investigations

Investigation is directed to confirm the diagnosis of MSCC and give information about the extent of the compression. Whole spine MRI is the investigation of choice. If MRI is contraindicated, CT whole spine could provide useful information. Assessment of underlying cancer status is important as development of MSCC could only be part of disseminated cancer progression [93].

#### Management

All patients with suspected MSCC should be admitted to hospital, placed on flat bed rest, log rolled, started on high-dose dexamethasone with PPI cover [93]. Once MSCC is diagnosed on imaging, spinal stability should be assessed and documented [93]. Discussion about definitive management will depend on prognosis based on cancer stage, response to treatment and patient’s fitness. Treatment options include surgery, radiotherapy or best supportive care. A definitive treatment plan—either radiotherapy or surgery—should be established as soon as possible, ideally within 24 h of the diagnosis. In pancreatic cancer, most of the patients have poor prognosis due to stage IV disease and most likely they will be treated with radiotherapy [94].

#### Complications of Treatment

Complication of treatment could be related to spinal surgery including acute and long-term surgical squealae. Acute complication such as bleeding during or shortly after surgery and iatrogenic inter-operative spinal cord injury are less common in specialised neurosurgical centres. Radiotherapy can cause local irritation to the skin and tissues surrounding spinal cord. Increased pain could be one of the early symptoms before gaining benefit from radiotherapy. Radiotherapy-induced myelitis is less common as most of the patients are on steroid at the time of treatment [94].

### Brain Metastases

#### Frequency

Brain metastases from gastrointestinal cancer is around 4%, and they are extremely rare (0.2%) in pancreatic cancer [95].

#### Symptoms

Headache in combination with nausea and vomiting are common symptoms as manifestation of increase intracranial pressure. Functional impairment and symptoms depend on the localisation of the metastases.

#### Investigations

Contrast-enhanced MRI is the preferred imaging study to provide information about site size and number of brain metastasis and also provide information about CSF flow and possible hydrocephalus. CT brain with contrast is also helpful but is less sensitive compared to MRI, and it should only be performed if MRI is not available or contraindicated. Assessment of underlying cancer status is important as development of brain metastases could only part of disseminated cancer progression [93].

MRI is helpful as it gives good information about meninges as some patients develop leptomeningeal disease. The prognosis of these patients is very poor with a survival of only a few months [93].

#### Management

Corticosteroids are useful in the initial management of symptoms suggesting increased intracranial pressure. Definitive treatment depends on the overall prognosis, extracranial disease control and patients’ fitness. Brain surgery and/or radiotherapy (whole brain or stereotactic) should be considered in patient with good prognosis and controlled extracranial disease. Patient with limited prognosis but remain functionally well (good PS) could be considered for systemic therapy [93].

## Conclusion

It is evident that patients with pancreaticobilliary malignancies are at increased risk of a wide variation of medical emergencies. Clinical knowledge, early recognition and collaboration with the relevant specialties are critical to manage these complications effectively. Understanding of patients’ prognosis and overall clinical condition would aid to tailor treatment options and manage expectations.
